# Nail-patella-syndrome in a young patient followed up over 10 years: relevance of the sagittal trochlear septum for patellofemoral pathology

**DOI:** 10.1051/sicotj/2016017

**Published:** 2016-05-31

**Authors:** Christian Konrads, Stephan Reppenhagen, Piet Plumhoff, Maximilian Rudert, Andre Steinert, Thomas Barthel

**Affiliations:** 1 Department of Orthopaedic Surgery, Koenig-Ludwig-Haus, Julius-Maximilians-University Wuerzburg Brettreichstr. 11 97074 Wuerzburg Germany

**Keywords:** Nail patella syndrome (NPS), Septum, Knee, Arthroscopy, Patella instability

## Abstract

*Introduction*: Nail-patella-syndrome (NPS) is a rare autosomal-dominant inherited disease with pathologies of nails, skeleton, kidneys, and eyes. Linkage to a mutated gene was found. It codes for the transcription-factor LMX1B. In most cases knees are symptomatic. Patients have hypoplastic patellae, which are laterally subluxated. In those individuals a sagittal trochlear fibrous septum was found, dividing the anterior knee-joint-space. In the literature the etiology and clinical significance of this anatomic abnormality is unclear. Based on clinical and intraoperative findings we developed a theory regarding knee pathology in nail-patella-syndrome. Successful treatment via early resection of the septum with sustained good outcome is presented.

*Methods*: In a symptomatic six-year-old boy with nail-patella-syndrome we resected the fibrous sagittal septum adherent to the trochlea femoris and we balanced the patella via lateral release and medial plication in both knee joints. We analyzed the clinical outcome of this procedure prospectively over 10 years.

*Results*: Postoperatively the hypoplastic patellae stayed centered and stable during further skeletal development. The patient was still pain free with normal range of motion of both operated knee joints after 10 years of follow-up.

*Discussion*: In patients with nail-patella-syndrome and a subluxated or dislocated patella we recommend diagnostics with magnetic-resonance-imaging and early surgical treatment via resection of the trochlear septum and soft-tissue-balancing of the patella. When the septum displaces the patella and prevents physiological articulation of the patella with the trochlea femoris, early septum resection is likely to be important for a good functional outcome and proper development of the patellofemoral joint during growth.

## Introduction

**Figure 1.**
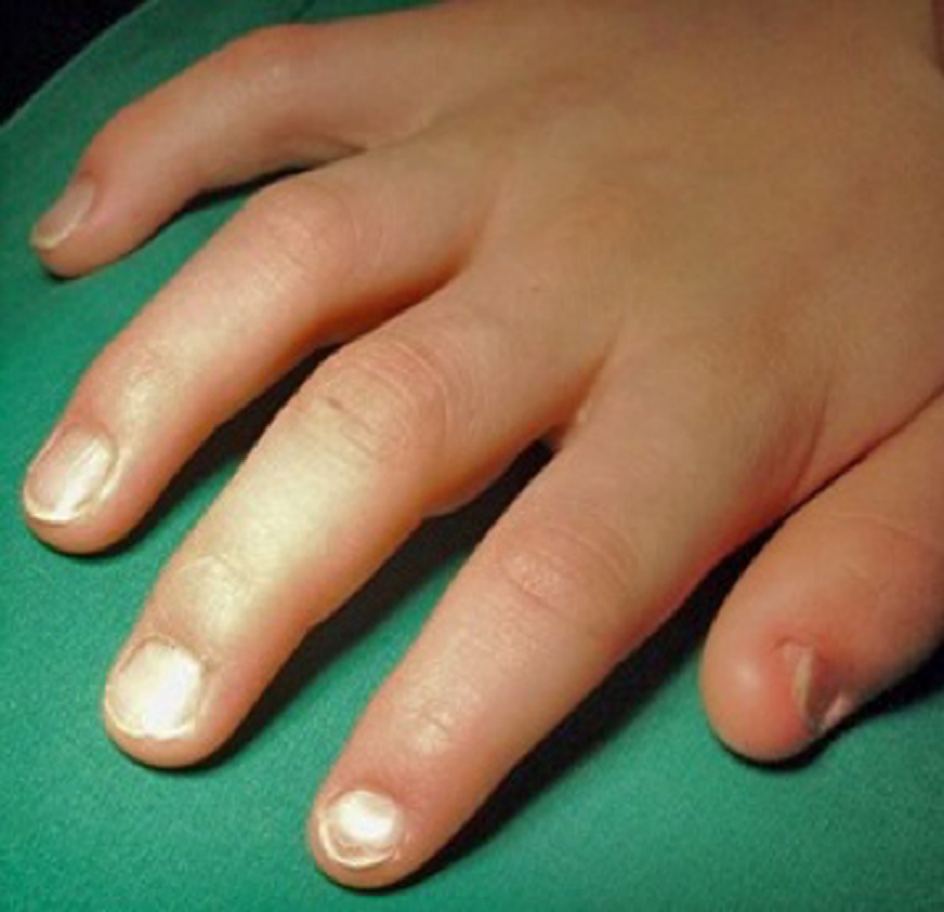
Typical hypoplastic finger nails in a six-year-old boy with nail-patella-syndrome. Note the decreasing pathology from first to fifth finger.

Nail-patella-syndrome (NPS) is also called hereditary onycho-osteo-dysplasia [1]. It is a rare autosomal dominant inherited disease of mesodermal and ectodermal tissue differentiation, with very high penetrance [1]. The incidence is reported between 1:50,000 and 1:250,000 [2]. There is a typical clinical tetrad of hypoplastic nails (Figure 1), hypoplastic patellae, radial head luxation, and posterior iliac horns (“Frog’s prongs”) [3]. Additionally, kidneys (basement membrane of glomeruli) and eyes (open angle glaucoma) can be involved with potential reduction of life expectancy limited by kidney function [4]. The syndrome was mentioned first by Chaterlain in 1820. Linkage to a mutated gene was found. It is located on the long arm of chromosome nine in region 9q34, close to an AB0 blood group gene. It codes for the transcription factor LMX1B. Loss-of-function mutations of LMX1B cause NPS [5].

Most NPS patients present with knee symptoms [6]. Regularly they have anterior knee pain and patellar instability in both knees. The hypoplastic patellae are generally laterally subluxated or dislocated. An intercondylar fibrous septum in the trochlea femoris was found previously [7, 8]. This strong septum was described to be sagittally oriented [7, 8]. In the literature, the etiology and clinical significance of this anatomic abnormality remain unclear to date [9].

Based on previous clinical and intraoperative findings in children and adults with NPS we developed a theory regarding knee pathology in this condition. Successful treatment via early resection of the septum with sustained good clinical and radiological outcome over 10 years of prospective follow-up is presented.

## Patient and methods

A six-year-old boy presented with anterior knee pain and laterally subluxated, hypoplastic patella in both knees. Fingernails were hypoplastic (Figure 1) and bilateral posterior iliac horns were present. Kidney function was normal and there was no eye pathology present. NPS had been diagnosed in the family. However, nobody in the family with NPS had kidney or eye involvement.

**Figure 2.**
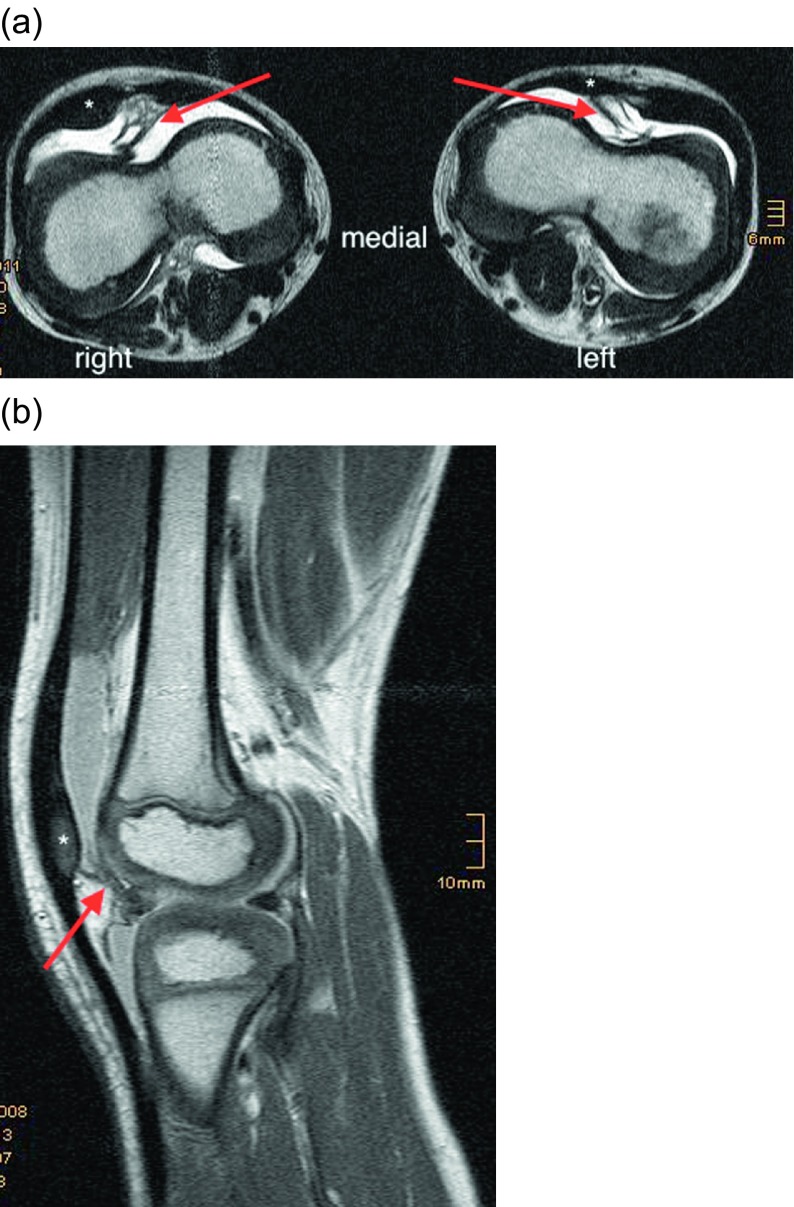
(a) Preoperative axial MRI of both knees in a six-year-old boy shows a deep femoral trochlea with a septum (red arrow), dividing the knee joint in a medial and lateral half and positioning the patella on the lateral side of the septum; patella ossification center (*). (b) Sagittal MRI through the lateral femoral condyle articulating with the patella ossification center (*); septum (red arrow).

We did magnetic resonance imaging (MRI) of both knees looking for a typical trochlear septum, which we had seen previously in other patients with NPS and knee complaints. MRI showed a large septum in a deep femoral trochlea in both knees of the patient (Figure 2a). The septum was sagittally oriented, adherent to the trochlear groove, reaching to the hoffa fat pad and anterior tibial plateau (Figure 2b).

**Figure 3.**
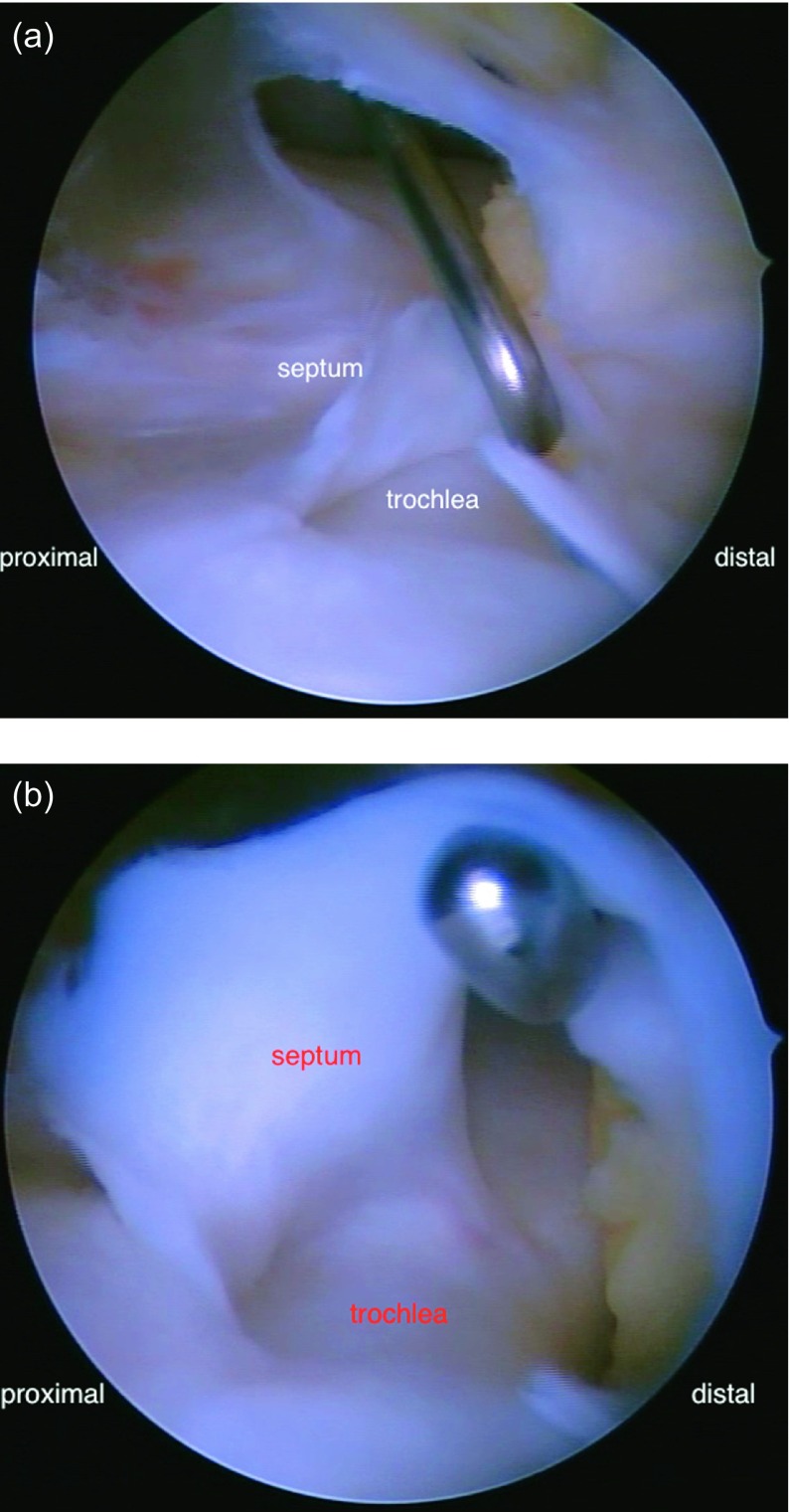
(a) Arthroscopy of the right knee from the anterolateral portal showed a strong sagittal septum in the femoral trochlea dividing the knee joint in a lateral and medial compartment. We perforated the septum with a hook via a high anteromedial portal and resected the septum. (b) The septum was adherent to the trochlear groove.

Together with the patient and his parents we decided for operative treatment of the right knee, which the patient reported to be more painful than the left knee. Under general anesthesia and tourniquet on the thigh at 300 mmHG we performed a diagnostic arthroscopy. We found a deep femoral trochlea with a strong sagittally oriented septum hindering the hypoplastic patella from articulating properly with the trochlea. The septum was partially adherent to the trochlear groove. The patella was subluxated laterally. The septum made it impossible to reach the medial aspect of the knee with the arthroscope via the anterolateral portal. We resected the whole septum arthroscopically as a first part of the treatment (Figures 3a and 3b).

After septum resection the patella did not perfectly center into the trochlea spontaneously. So we decided for proximal patella realignment with open medial plication in combination with a moderate lateral soft tissue release. With this procedure we achieved a nicely centered and stable patella. Postoperatively range of motion was limited to 0/0/90° for four weeks. At the time of six weeks follow-up visit, patient and parents asked for surgical therapy of the contralateral left knee. Therefore, three months after operation of the right knee, we undertook surgery of the left knee. We found the same intraarticular pathology in the left knee and performed a similar surgical procedure like we did in the right knee. Again we reached a well-balanced patellofemoral joint. Follow-up examinations were conducted after six weeks and yearly thereafter for over 10 years comprising documentation of pain (visual analog scale; VAS), range of motion (ROM), and joint stability. At the last follow-up examination, the patient was 17 years old. This was 126 months after surgery of the right knee and 123 months after surgery of the left knee.

## Results

**Figure 4.**
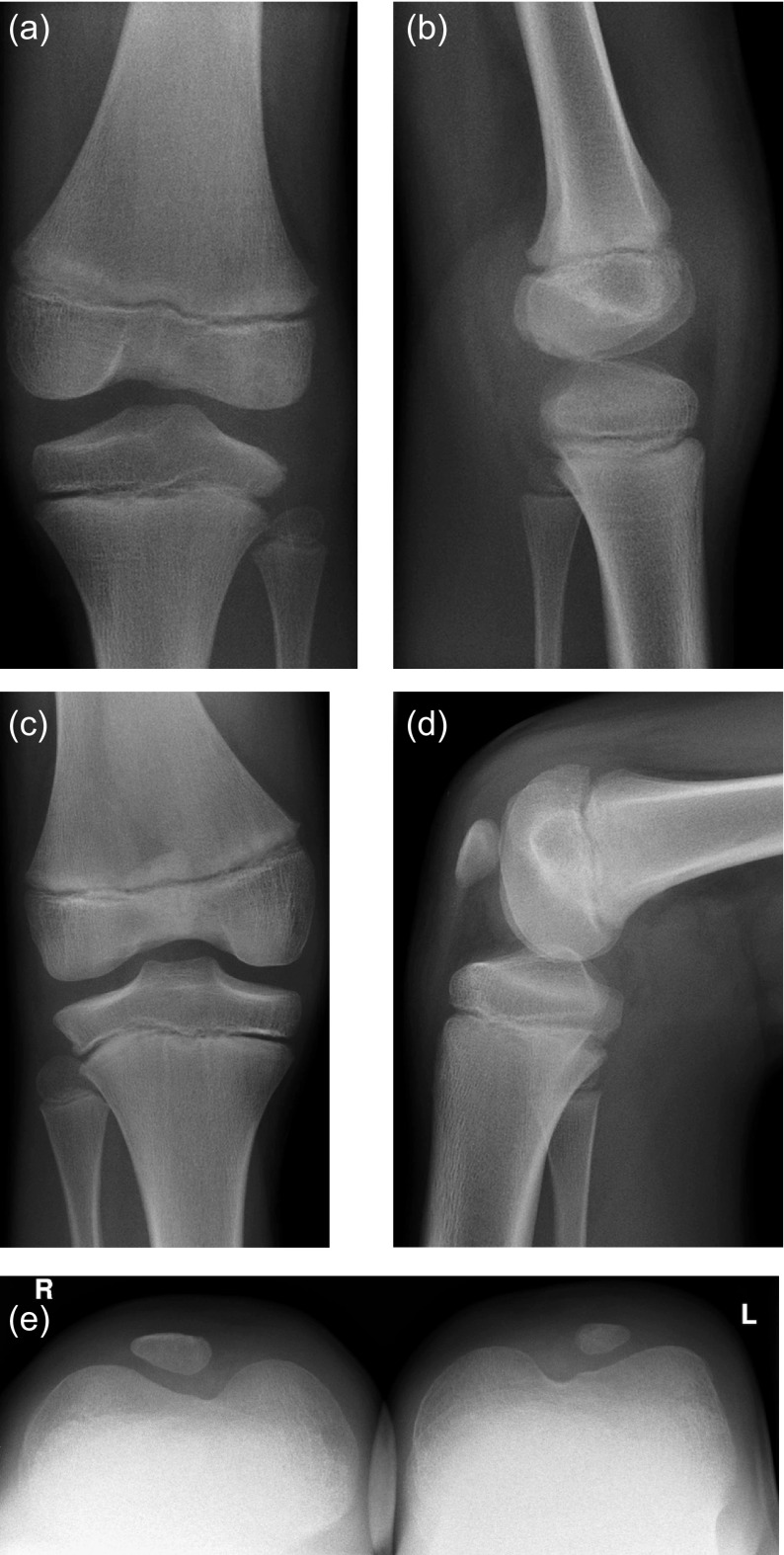
(a, b) Radiological follow-up two years postoperatively at age of eight no patella is seen on X-rays. (c–e) Six years postoperatively at age of 12 patellae are hypoplastic but centered and trochleae are deep.

After surgical treatment of both symptomatic knees with septum resection and proximal patella realignment, there were no complications or revisions during a follow-up of over 10 years. Postoperatively, the hypoplastic patellae stayed centered and stable in both knees during further skeletal development upon clinical and radiographical examinations (Figures 4a–4e). The patient was pain free with full range of motion (0/0/140°) of both operated knee joints 10 years postoperatively. At this time, he was performing active sports by playing in a hockey team several times a week.

## Discussion

In patients with NPS, hypoplastic and laterally subluxated or dislocated patellae and anterior knee pain are common [6]. In those patients a thick septum in the femoral trochlea was described earlier [10, 11]. In the literature, the etiology and relevance of this septum are not absolutely clear [9]. The LMX1B transcription factor, which is affected in NPS patients, plays an important role in early mesenchymal development. The persistent septum may be a flaw of apoptotic processes necessary for appropriate formation of the anterior knee joint cavity. Recurrence of the anterior knee septum and symptoms after septum resection in one of two cases were reported by others [9].

According to our findings in children and adults diagnosed with NPS presenting typical knee pathologies, we think that the thick sagittally oriented septum in the femoral trochlea leads to pathognomonic anatomic developments during growth: (1) the trochlea femoris becomes deep and scalloping; (2) the patella remains on the lateral side of the septum and cannot center into the trochlea. During growth the septum remains centrally within the trochlea groove and thereby displaces the patella laterally. This leads to elongation of the passive medial stabilizers like the medial patellofemoral ligament (MPFL). Simultaneously the lateral soft tissues like the lateral retinaculum stay shortened. The older the patient the more complex the soft tissue pathologies are. This is due to adaptation of the passive stabilizers to the severely lateralized patella during skeletal development. Altered patellofemoral biomechanics with a lateralized patella may lead to early symptomatic patellofemoral arthritis.

Diagnostics with MRI is important. In young children, the patellar ossification center is not seen on X-rays (Figures 4a and 4b). MRI shows the patellar anlage and the typical septum (Figure 2). Arthro-MRI depicts the septum even better, which divides the proximal recessus into a medial and lateral compartment. We recommend early arthroscopic septum resection before school enrollment of the patient. If necessary, proximal realignment of the patella should be performed simultaneously.

After complete septum resection in combination with proximal patella realignment in both knees of a six-year-old boy with NPS, the patient had a very good outcome and stayed active through adolescence over a follow-up of over 10 years. As patellae in both knees stayed centered and stable there is no note of recurrence of the septum, which was mechanically compromising the patellofemoral joint preoperatively.

Further research and mid- to long-time follow-up studies of more patients after early operative therapy of the typical knee pathology in NPS are needed to identify potential problems and recurrence rate regarding the described surgical management.

## Conclusions

In patients with nail-patella-syndrome and a subluxated or luxated patella, we recommend diagnostics with MRI and early surgical treatment via resection of the sagittal septum and additional soft tissue balancing of the patella, if needed. Because the septum displaces the patella and prevents engagement of the patella with the trochlea femoris, probably early septum resection is important for good function and development of the patellofemoral joint during growth. We demonstrated successful treatment of both knees in a six-year-old boy with very satisfying outcome over a follow-up of over 10 years.

## Conflict of interest

The authors declare that they have no conflict of interest.
